# Development and validation of an artificial intelligence model for predicting de novo distant bone metastasis in breast cancer: a dual-center study

**DOI:** 10.1186/s12905-024-03264-z

**Published:** 2024-08-05

**Authors:** Wen-hai Zhang, Yang Tan, Zhen Huang, Qi-xing Tan, Yue-mei Zhang, Chang-yuan Wei

**Affiliations:** 1 Department of Breast Surgery, Guangxi Medical University Cancer Hospital, 71 Hedi Road, Nanning, Guangxi Zhuang Autonomous Region 530021 China; 2 Department of Pathology, The First Affiliated Hospital of Guangxi Medical University, Nanning, Guangxi China

**Keywords:** AI, LightGBM, Bone metastasis, Breast cancer, Dual-center

## Abstract

**Objective:**

Breast cancer has become the most prevalent malignant tumor in women, and the occurrence of distant metastasis signifies a poor prognosis. Utilizing predictive models to forecast distant metastasis in breast cancer presents a novel approach. This study aims to utilize readily available clinical data and advanced machine learning algorithms to establish an accurate clinical prediction model. The overall objective is to provide effective decision support for clinicians.

**Methods:**

Data from 239 patients from two centers were analyzed, focusing on clinical blood biomarkers (tumor markers, liver and kidney function, lipid profile, cardiovascular markers). Spearman correlation and the least absolute shrinkage and selection operator regression were employed for feature dimension reduction. A predictive model was built using LightGBM and validated in training, testing, and external validation cohorts. Feature importance correlation analysis was conducted on the clinical model and the comprehensive model, followed by univariate and multivariate regression analysis of these features.

**Results:**

Through internal and external validation, we constructed a LightGBM model to predict de novo bone metastasis in newly diagnosed breast cancer patients. The area under the receiver operating characteristic curve values of this model in the training, internal validation test, and external validation test1 cohorts were 0.945, 0.892, and 0.908, respectively. Our validation results indicate that the model exhibits high sensitivity, specificity, and accuracy, making it the most accurate model for predicting bone metastasis in breast cancer patients. Carcinoembryonic Antigen, creatine kinase, albumin-globulin ratio, Apolipoprotein B, and Cancer Antigen 153 (CA153) play crucial roles in the model’s predictions. Lipoprotein a, CA153, gamma-glutamyl transferase, α-Hydroxybutyrate dehydrogenase, alkaline phosphatase, and creatine kinase are positively correlated with breast cancer bone metastasis, while white blood cell ratio and total cholesterol are negatively correlated.

**Conclusion:**

This study successfully utilized clinical blood biomarkers to construct an artificial intelligence model for predicting distant metastasis in breast cancer, demonstrating high accuracy. This suggests potential clinical utility in predicting and identifying distant metastasis in breast cancer. These findings underscore the potential prospect of developing economically efficient and readily accessible predictive tools in clinical oncology.

**Supplementary Information:**

The online version contains supplementary material available at 10.1186/s12905-024-03264-z.

## Background

As the most common malignancy in women, breast cancer (BC) accounts for 30% of all cancers in females [1]. While its mortality rate ranks fourth, the number of new deaths is increasing most significantly [2]. This may be attributed to a continual decline in fertility rates and an increase in body weight [3]. In China, there are over 410,000 new cases of breast cancer and over 110,000 related deaths annually [4]. In recent years, the incidence of distant metastasis recurrence in BC patients remains high, serving as an adverse prognostic indicator.

For distant metastasis, the bone is the most common site, with over 60% of BC patients experiencing bone metastasis [5]. In Western countries, approximately 3.5–10% of all newly diagnosed breast cancer patients are diagnosed with distant metastasis [6]. For initially diagnosed metastatic breast cancer, this implies fewer treatment options and shorter survival times. Stage IV breast cancer is also a significant public health concern in many developed countries, exacerbated in resource-poor areas due to a lack of screening programs and early detection methods, resulting in many patients presenting with metastases at diagnosis [7]. Furthermore, while the 5-year overall survival rate for BC patients without metastasis exceeds 80% [8], distant metastasis significantly reduces this rate to only around 25% [9]. Notably, the 5-year overall survival rate for bone metastasis (BM) is even lower, at only about 22.8% [10]. Therefore, early identification of breast cancer bone metastasis has become a crucial issue that clinicians must address.

Currently, the identification and diagnosis of bone metastasis primarily rely on imaging techniques such as X-rays, bone scintigraphy, computed tomography, magnetic resonance imaging (MRI), and positron emission tomography-computed tomography. Among these, X-ray examination is the most widely used and cost-effective method in China. However, despite its high specificity, X-rays have low sensitivity, making it difficult to detect early metastatic lesions [11]. Moreover, other imaging tests suffer from unequal distribution of medical resources, equipment limitations, and high costs. Even in some developed regions, over-testing may occur without prior evaluation, leading to prolonged average hospital stays and increased hospital costs.

For clinicians, treating diseases requires individualization, advocating for precision medicine. Currently, precision medicine has evolved around four concepts: predictiveness, personalization, prevention, and participation [12]. Big data analysis techniques are becoming essential in clinical practice [13], indicating the need to utilize advanced technology to analyze vast amounts of medical data and provide recommendations for individualized treatment. Many studies have used machine learning techniques to investigate clinical risk factors associated with cancer metastasis to achieve early detection [14–16]. In recent years, several breast cancer bone metastasis (BCBM) prediction models have been developed using factors such as age, gender, race, treatment, and grade as predictive factors [17, 18]. However, these models still have specific areas for improvement in practicality and accuracy. This study aims to establish a more accurate clinical model, incorporating as many effective variables as possible.

Regarding model development, although nomograms are currently the most commonly used predictive models, machine learning is increasingly favored by medical professionals for its practicality, innovation, and accuracy. This study is based on common inpatient laboratory indicators in the real world, requiring no related pathological examination or imaging assessment, thus reducing the threshold for model establishment. Through horizontal comparison of multiple indicators, a reliable BCBM prediction model has been developed.

Ultimately, our goal is to stratify the risk of bone metastasis in breast cancer patients, assisting clinicians, especially primary breast specialists, in making decisions to alleviate unnecessary medical burdens on patients and greatly improve their quality of life.

## Materials and methods

### Patient population

This retrospective study included data from two medical centers, approved by the institutional review boards of both centers. Inclusion criteria were as follows: (1) clear diagnosis of primary breast cancer with de novo bone metastasis; (2) completion of clinical blood biomarker testing before treatment (radiotherapy or chemotherapy) or surgical resection; (3) no history of hypertension, diabetes, or hyperlipidemia; (4) no history of abnormal blood indicators related to liver, kidney, or cardiovascular function; (5) no history of other diseases. Exclusion criteria were as follows: (1) occurrence of distant metastasis after treatment (surgical resection or chemotherapy); (2) incomplete clinical blood biomarker data, including tumor markers (Alpha-fetoprotein (AFP), Carcinoembryonic Antigen (CEA), Cancer Antigen 125 (CA125), Cancer Antigen 153 (CA153), and Cancer Antigen 199 (CA199)), liver function tests, kidney function tests, lipid profile, or cardiovascular function tests; (3) age less than 18 years old; (4) occurrence of metastasis in sites other than bones.

**Fig. 1:**
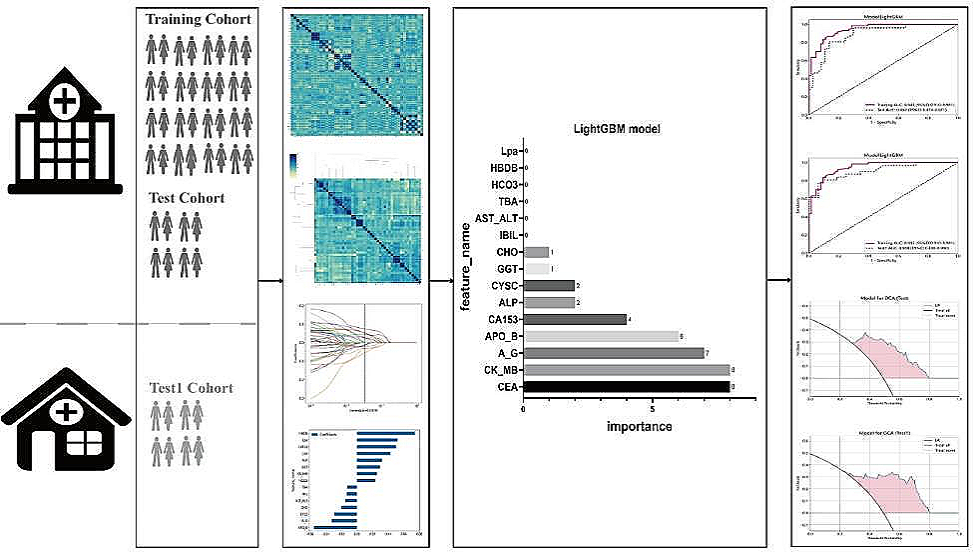
The workflow of LightGBM model in this study

The study involved breast cancer cases from two research centers. One center included 176 cases, randomly divided at an 8:2 ratio into training (123 cases) and test (53 cases) cohorts. Another center provided 63 cases as an external validation cohort. The internal validation cohort (test cohort) consisted of data from the same medical center as the training cohort, characterized by similar clinical treatment processes and data collection standards, which facilitated the evaluation of the model’s robustness and performance in similar clinical environments. The external validation cohort came from a geographically proximate but different medical center, validating the model’s generalizability across different institutions and patient populations. The purpose of selecting these two validation cohorts was to comprehensively assess the reliability and applicability of the model under diverse conditions. The distribution details of the study are provided in Table 1. The workflow of the model in this study is illustrated in Fig. 1.

**Table 1:** Clinical blood markers in the training, test, and Test1 cohorts

Characteristics	Training cohort (*n* = 123)				Test cohort (*n* = 53)				Test1 cohort (*n* = 63)		
	Non-distant metastasis (*n* = 63)	Distant metastasis (*n* = 60)	*P* value		Non-distant metastasis (*n* = 27)	Distant metastasis (*n* = 26)	*P* value		Non-distant metastasis (*n* = 32)	Distant metastasis (*n* = 31)	*P* value
Age (years), mean ± SD	49.06 ± 9.77	50.95 ± 11.03	0.317		48.59 ± 8.01	52.58 ± 10.01	0.115		51.50 ± 11.65	50.58 ± 10.31	0.742
Weight (kg), mean ± SD	58.66 ± 7.89	55.87 ± 8.60	0.063		59.48 ± 10.18	55.67 ± 10.27	0.181		58.42 ± 10.48	52.75 ± 7.56	0.017
CEA (ng/ml), mean ± SD	3.12 ± 1.74	27.17 ± 184.86	0.001		3.71 ± 6.31	45.27 ± 93.81	0.026		2.73 ± 3.23	238.82 ± 856.93	0.124
AFP (ng/ml), mean ± SD	32.67 ± 108.52	57.38 ± 135.88	0.304		2.28 ± 0.81	2.96 ± 2.35	0.167		2.32 ± 0.97	3.81 ± 4.01	0.045
CA125 (U/ml), mean ± SD	16.02 ± 15.07	95.41 ± 163.32	0.266		16.55 ± 9.14	68.84 ± 191.57	0.163		20.53 ± 20.05	226.60 ± 594.40	0.055
CA153 (U/ml), mean ± SD	12.21 ± 21.91	23.22 ± 60.36	< 0.001		39.59 ± 128.51	97.47 ± 159.52	0.151		14.75 ± 9.88	210.74 ± 359.05	0.003
CA199 (U/ml), mean ± SD	12.51 ± 5.36	11.80 ± 4.10	0.177		11.49 ± 19.30	25.13 ± 37.02	0.097		10.73 ± 13.97	96.68 ± 446.91	0.281
TBIL (µ mol/L), mean ± SD	3.77 ± 1.54	3.96 ± 1.37	0.410		13.13 ± 3.95	10.23 ± 3.24	0.005		13.68 ± 4.45	19.25 ± 35.12	0.377
DBIL (µ mol/L), mean ± SD	8.74 ± 3.96	7.49 ± 2.06	0.483		4.11 ± 1.38	3.35 ± 1.00	0.026		3.44 ± 1.37	8.60 ± 22.69	0.204
IBIL (µ mol/L), mean ± SD	69.79 ± 6.02	69.13 ± 6.53	0.031		9.01 ± 2.75	6.87 ± 2.45	0.004		10.24 ± 3.45	10.65 ± 12.80	0.860
TP (g/L), mean ± SD	40.31 ± 5.54	38.16 ± 5.59	0.563		67.99 ± 6.29	70.08 ± 7.85	0.290		71.09 ± 6.47	72.17 ± 5.87	0.489
ALB (g/L), mean ± SD	28.55 ± 4.70	30.37 ± 4.99	0.035		40.19 ± 2.54	38.35 ± 4.26	0.060		39.87 ± 2.87	37.82 ± 4.97	0.049
GLO (g/L), mean ± SD	1.45 ± 0.21	1.30 ± 0.26	0.040		27.80 ± 4.90	31.35 ± 5.81	0.020		31.23 ± 5.24	34.35 ± 5.96	0.031
A_G (Ratio), mean ± SD	237.53 ± 48.47	219.49 ± 96.29	< 0.001		1.48 ± 0.20	1.24 ± 0.22	< 0.001		1.30 ± 0.23	1.13 ± 0.27	0.008
GGT (U/L), mean ± SD	17.00 ± 10.06	25.83 ± 24.11	0.189		244.51 ± 49.26	209.85 ± 48.36	0.013		257.54 ± 37.68	196.58 ± 68.65	< 0.001
TBA (µ mol/L), mean ± SD	28.02 ± 13.56	37.20 ± 29.15	0.009		16.22 ± 6.01	24.62 ± 21.31	0.055		14.25 ± 5.29	35.42 ± 71.70	0.101
PA (mg/L), mean ± SD	1.90 ± 0.94	1.74 ± 0.71	0.026		23.26 ± 5.56	36.12 ± 21.19	0.004		21.56 ± 8.08	86.55 ± 259.90	0.162
AST (U/L), mean ± SD	66.56 ± 22.22	106.66 ± 96.23	0.289		2.18 ± 3.38	1.86 ± 0.93	0.644		1.60 ± 0.55	2.19 ± 1.43	0.032
ALT (U/L), mean ± SD	178.16 ± 49.26	224.22 ± 106.54	0.002		66.30 ± 21.73	123.19 ± 86.73	0.002		62.56 ± 20.19	148.06 ± 117.46	< 0.001
AST_ALT (Ratio), mean ± SD	21.33 ± 15.30	37.93 ± 37.15	0.002		178.37 ± 65.42	281.65 ± 250.64	0.044		149.81 ± 28.89	260.40 ± 172.26	< 0.001
ALP (U/L), mean ± SD	8757.29 ± 2285.08	8513.48 ± 2086.49	0.001		20.39 ± 10.44	42.77 ± 64.34	0.080		20.59 ± 11.47	95.26 ± 230.50	0.072
CHE (U/L), mean ± SD	6.41 ± 11.53	4.62 ± 3.70	0.538		9357.44 ± 1848.61	8526.35 ± 2439.21	0.167		8807.53 ± 1700.66	7805.23 ± 2451.91	0.063
UREA (mmol/L), mean ± SD	10.87 ± 50.36	5.21 ± 2.86	0.255		6.38 ± 7.63	7.03 ± 9.74	0.785		4.71 ± 3.41	18.09 ± 45.47	0.102
CREA (µ mol/L), mean ± SD	64.67 ± 26.93	66.35 ± 31.21	0.386		4.87 ± 1.68	5.27 ± 2.52	0.495		4.36 ± 1.12	4.36 ± 1.49	0.992
UA (µ mol/L), mean ± SD	318.97 ± 85.41	315.11 ± 96.83	0.749		60.26 ± 11.94	61.96 ± 17.55	0.680		61.03 ± 10.44	59.97 ± 15.99	0.755
HCO3 (mmol/L), mean ± SD	25.22 ± 3.28	25.78 ± 2.78	0.815		298.59 ± 111.03	310.46 ± 105.17	0.691		311.66 ± 69.55	323.77 ± 120.77	0.626
Ccr (ml/min), mean ± SD	3.91 ± 0.28	3.90 ± 0.43	0.310		25.96 ± 2.19	26.31 ± 2.17	0.568		24.93 ± 2.71	24.94 ± 2.45	0.999
CYSC (mg/L), mean ± SD	140.78 ± 5.68	140.97 ± 2.95	0.886		4.05 ± 0.38	4.02 ± 0.42	0.793		4.00 ± 0.33	3.94 ± 0.53	0.570
K (mmol/L), mean ± SD	101.71 ± 11.46	98.73 ± 18.54	0.819		135.90 ± 24.45	140.78 ± 2.14	0.316		139.54 ± 2.00	139.49 ± 2.30	0.931
Na (mmol/L), mean ± SD	2.28 ± 0.28	2.32 ± 0.30	0.282		102.33 ± 2.76	101.69 ± 2.51	0.381		106.02 ± 2.26	104.78 ± 3.06	0.072
Cl (mmol/L), mean ± SD	1.00 ± 0.12	0.98 ± 0.13	0.460		2.27 ± 0.15	2.32 ± 0.15	0.273		2.26 ± 0.08	2.26 ± 0.40	0.964
Ca (mmol/L), mean ± SD	2.47 ± 10.95	1.17 ± 0.22	0.333		1.00 ± 0.11	0.95 ± 0.16	0.140		0.89 ± 0.20	0.86 ± 0.09	0.374
Mg (mmol/L), mean ± SD	0.90 ± 0.31	0.91 ± 0.36	0.358		1.11 ± 0.21	1.14 ± 0.22	0.631		1.12 ± 0.14	1.26 ± 0.23	0.006
PHO (mmol/L), mean ± SD	93.26 ± 24.34	88.88 ± 28.15	0.969		0.82 ± 0.23	0.92 ± 0.25	0.156		0.82 ± 0.14	0.88 ± 0.22	0.187
CK (U/L), mean ± SD	83.97 ± 30.60	77.38 ± 43.75	0.357		100.76 ± 16.24	87.62 ± 28.63	0.044		96.59 ± 14.58	91.89 ± 19.88	0.288
CK_MB (U/L), mean ± SD	9.06 ± 4.73	19.83 ± 31.34	0.333		99.11 ± 83.20	72.69 ± 23.92	0.125		80.62 ± 62.88	69.84 ± 28.89	0.388
LDH (U/L), mean ± SD	124.97 ± 34.44	166.52 ± 75.81	0.008		11.26 ± 5.21	31.38 ± 34.21	0.004		13.34 ± 2.89	23.84 ± 15.25	< 0.001
HBDB (U/L), mean ± SD	5.37 ± 1.13	4.86 ± 0.88	< 0.001		126.37 ± 28.97	209.62 ± 218.05	0.055		108.47 ± 20.83	189.60 ± 165.41	0.008
CHO (mmol/L), mean ± SD	1.45 ± 0.92	1.40 ± 0.91	0.007		5.23 ± 1.21	5.08 ± 0.98	0.616		5.19 ± 0.99	4.79 ± 0.95	0.108
TG (mmol/L), mean ± SD	1.37 ± 0.39	1.30 ± 0.33	0.767		1.48 ± 1.03	1.55 ± 1.18	0.804		1.39 ± 0.93	1.52 ± 0.91	0.555
HDL_C (mmol/L), mean ± SD	3.11 ± 0.95	2.83 ± 0.74	0.271		1.32 ± 0.30	1.45 ± 0.48	0.235		1.41 ± 0.26	1.29 ± 0.29	0.084
LDL_C (mmol/L), mean ± SD	1.33 ± 0.20	1.24 ± 0.20	0.069		3.13 ± 0.97	2.90 ± 0.83	0.356		3.15 ± 0.84	2.83 ± 0.80	0.127
APO_A1 (g/L), mean ± SD	1.00 ± 0.29	0.87 ± 0.23	0.020		1.28 ± 0.21	1.38 ± 0.30	0.175		1.32 ± 0.22	1.24 ± 0.20	0.105
APO_B (g/L), mean ± SD	1.47 ± 0.44	1.49 ± 0.38	0.008		1.02 ± 0.46	3.73 ± 13.93	0.317		0.86 ± 0.22	0.82 ± 0.18	0.449
A1_B (Ratio), mean ± SD	198.10 ± 194.21	297.68 ± 253.21	0.776		1.46 ± 0.50	1.68 ± 0.81	0.243		1.65 ± 0.49	1.62 ± 0.38	0.792
Lpa (mg/L), mean ± SD	243.74 ± 236.81	340.65 ± 302.95	0.016		239.19 ± 209.69	443.31 ± 380.25	0.019		271.19 ± 265.21	186.85 ± 150.34	0.127
Pathological diagnosis*, n (%)			—				—				—
Infiltrating duct carcinoma, NOS	62(98.41)	56(93.33)			25(92.59)	24(92.31)			32(100.00)	28(90.32)	
Infiltrating lobular carcinoma, NOS	0(0.00)	3(5.00)			1(3.70)	2(7.69)			0(0.00)	2(6.45)	
Invasive papillary carcinoma	0(0.00)	1(1.67)			1(3.70)	0(0.00)			0(0.00)	1(3.23)	
Invasive micropapillary carcinoma	1(1.59)	0(0.00)			0(0.00)	0(0.00)			0(0.00)	0(0.00)	
Lymph node metastasis*, n (%)			—				—				—
Absent	32(50.79)	4(6.67)			13(48.15)	0(0.00)			24(75.00)	2(6.45)	
Present	31(49.21)	56(93.33)			14(51.85)	26(100.00)			8(25.00)	29(93.55)	

*SD* Standard deviation; *AFP* Alpha-Fetoprotein; *CEA* Carcinoembryonic Antigen; *CA125* Carbohydrate Antigen125; *CA153* Carbohydrate Antigen153; *CA199* Carbohydrate Antigen 199; *TBIL* total bilirubin; *DBIL* direct bilirubin; *IBIL* indirect bilirubin; *TP* total protein; *ALB* albumin; *GLO* globulin; *A_G (Ratio)* albumin-globulin ratio; *GGT* γ-glutamyl transferase; *TBA* total bile acids; *PA* pre-albumin; *AST* aspartate aminotransferase; *ALT* alanine aminotransferase; *AST_ALT (Ratio)* AST/ALT ratio; *ALP* alkaline phosphatase; *CHE* cholinesterase; *UREA* urea; *CREA* creatinine; *UA* uric acid; *HCO3* blood bicarbonate concentration; *Ccr* endogenous creatinine clearance rate; *CYSC* cysteine protease inhibitor C; *K* potassium ion; *Na* sodium ion; *Cl* chloride ion; *Ca* calcium ion; *Mg* magnesium ion; *PHO* inorganic phosphate; *CK* creatine kinase; *CK_MB* creatine kinase isoenzyme MB; *LDH* lactate dehydrogenase; *α-HBDB* α-hydroxybutyrate dehydrogenase; *CHO* total cholesterol; *TG* triglycerides; *HDL_C* high-density lipoprotein cholesterol; *LDL_C* low-density lipoprotein cholesterol; *APO_A1* Apolipoprotein A1; *APO_B* Apolipoprotein B; *A1_B (Ratio)* Apolipoprotein A1/Apolipoprotein B ratio; *Lpa* lipoprotein (a)

### Feature extraction and selection

The features included from clinical blood biomarkers comprised tumor markers (AFP; CEA; CA125; CA153; CA199), liver function indicators (total bilirubin, direct bilirubin, indirect bilirubin, total protein, albumin, globulin, albumin-globulin ratio, gamma-glutamyl transferase, prealbumin, aspartate transaminase (AST), alanine transaminase (ALT), AST/ALT ratio, alkaline phosphatase, cholinesterase, and total bile acid), kidney function indicators (urea, creatinine, uric acid, blood bicarbonate concentration, cystatin C, potassium ion, sodium ion, chloride ion, calcium ion, and inorganic phosphorus), lipid profile (total cholesterol, triglycerides, high-density lipoprotein cholesterol, low-density lipoprotein cholesterol, apolipoprotein A1, apolipoprotein B, A1/B ratio, and lipoprotein (a)), and cardiovascular function indicators (creatine kinase, creatine kinase isoenzyme MB (CK-MB), lactate dehydrogenase (LDH), and α-Hydroxybutyrate dehydrogenase (α-HBDH)).

All extracted features underwent the following operations: first, Z-Score standardization (mean = 0, standard deviation = 1) was applied to normalize each feature, preprocessing the data to fit a standard normal distribution. Then, statistical analysis was conducted using Spearman rank correlation coefficient (ρ) to measure the correlation between two variables. ρ is a non-parametric statistical measure of the strength of a monotonic relationship between two variables. When ρ approaches 1 or -1, it indicates a strong correlation between the variables. We chose ρ > 0.9 as the threshold for high correlation. High correlation means that the variables exhibit very consistent trends, which can lead to multicollinearity issues. Highly correlated features can introduce redundant information, increase model complexity, and affect the stability and interpretability of the model. When the Spearman correlation coefficient between features was > 0.9, one of the features was retained, as keeping only one variable with a correlation coefficient greater than 0.9 helps reduce redundancy and improve the model’s generalizability.

Finally, feature dimension reduction was conducted using L1 regularization of the Least Absolute Shrinkage and Selection Operator (LASSO) regression. The LASSO method penalizes the absolute values of regression coefficients, thereby inducing some coefficients to be zero, facilitating feature selection and generating a sparse model. In LASSO regression, the choice of lambda (λ) is critical as it controls the strength of the penalty applied to regression coefficients. A higher lambda increases the penalty, leading more coefficients to shrink to zero, simplifying the model but posing a risk of underfitting. Conversely, a lower lambda reduces the penalty, potentially including more features but risking overfitting to the training data. Our 10-fold cross-validation process helped identify a lambda value that generalizes well to unseen data. We selected the lambda parameter by performing 10-fold cross-validation on the training set, choosing the value that minimized mean squared error. This approach ensures an optimal balance between model complexity and predictive performance, aiding in preventing overfitting.

### Development and validation of models

In this study, the LightGBM machine learning algorithm was employed to construct models for breast cancer with and without bone metastasis as binary outcome variables, using the selected features for dimension reduction. Model construction was completed based on 5-fold cross-validation in the training set. After model construction, validation was conducted in both internal and external testing cohorts. Model performance was evaluated using metrics such as the area under the receiver operating characteristic curve (AUC), accuracy, sensitivity, specificity, positive predictive value, and negative predictive value. Subsequently, decision curve analysis (DCA) was performed to reflect the net benefit at different threshold probabilities in the training and internal and external validation cohorts, evaluating the clinical efficiency of the model.

### Statistical analysis

Clinical baseline features were analyzed using t-tests, chi-square tests, or Fisher’s exact tests with SPSS software (version 25.0, IBM). The t-test was used for continuous variables with homogeneity of variance, represented as x ± s, while the chi-square test or Fisher’s exact test was used for categorical variables, represented as ratios. A two-tailed p-value < 0.05 indicated statistical significance. Spearman rank correlation tests, z-score normalization, univariate regression analysis, multivariate regression analysis, output of feature importance for LightGBM models, and LASSO regression analysis were performed using Python software (version 3.7.17; http://www.python.org). ROC curves and clinical decision curves were also plotted.

## Results

### Patient characteristics

This study involved a total of 239 female breast cancer patients from two research centers. One center contributed 123 cases to the training cohort, 53 cases to the testing cohort, and the other center provided 63 cases for the test1 cohort. In the baseline characteristic analysis of the study population, statistically significant differences were observed in one, two, or three cohorts for various blood biomarkers including CEA, CA153, total bilirubin, direct bilirubin, indirect bilirubin, albumin, globulin, albumin/globulin ratio, gamma-glutamyl transferase, total bile acid, prealbumin, aspartate transaminase, alanine transaminase, aspartate/alanine ratio, alkaline phosphatase, magnesium ion, creatine kinase, LDH, α-HBDH, total cholesterol, apolipoprotein A1, apolipoprotein B, and lipoprotein a. A summary of patient clinical blood biomarker features is presented in Table 1.

### Feature selection

**Fig. 2:**
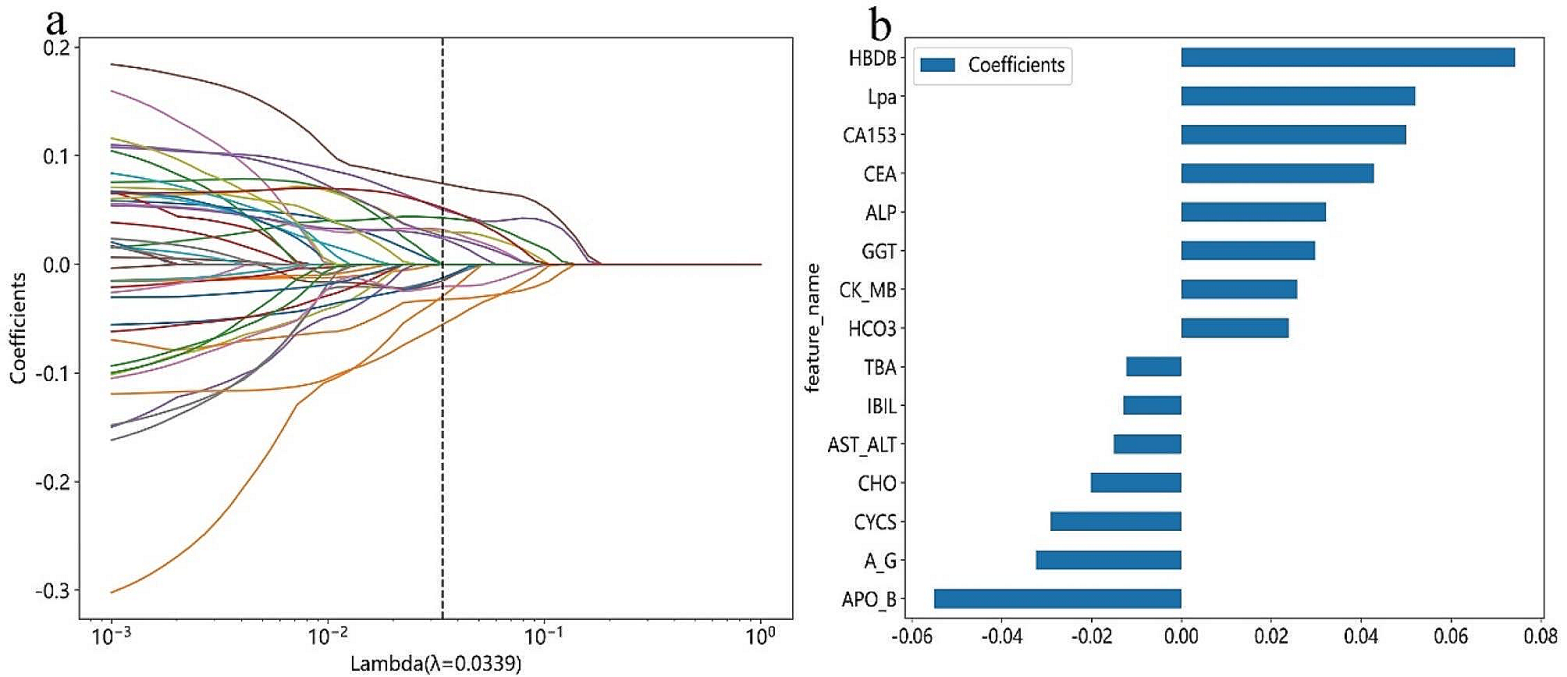
Illustrates the process of feature selection using the least absolute shrinkage and selection operator (LASSO) regression model. (**a**) LASSO coefficients for different λ values, where vertical dashed lines indicate the number of features corresponding to the optimal λ value. (**b**) After feature selection using LASSO regression, the nonzero coefficient features are showed

Feature data were normalized, and features with a Spearman correlation coefficient > 0.9 were retained. The heatmap illustrating the correlation analysis of features is shown in Supplementary Fig. 1. Dimension reduction was performed by eliminating features with zero coefficients using LASSO regression. The optimal λ value was determined based on the minimum mean squared error, and the Lasso regression model was fitted accordingly (Fig. 2a). After feature dimension reduction, 15 features were selected for each cohort (Fig. 2b).

#### Model construction and validation

**Fig. 3:**
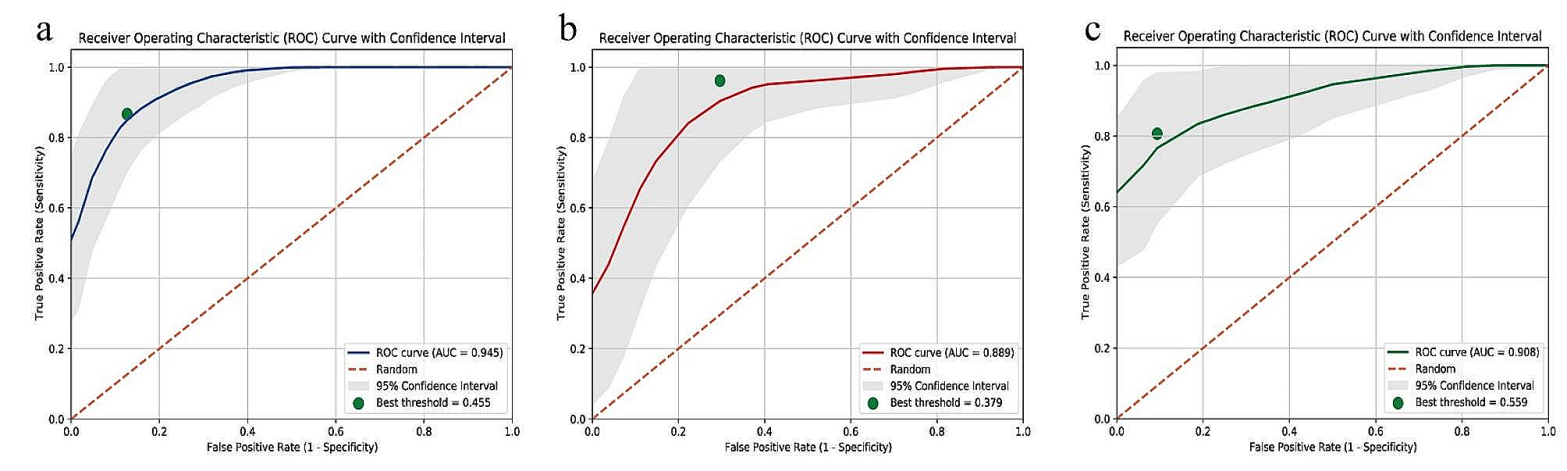
Evaluation of Receiver Operating Characteristic curves for the LightGBM models constructed in both the training (**a**), test (**b**) and test1 (**c**) cohorts were presented

The LightGBM machine learning algorithm was utilized to construct predictive models for breast cancer bone metastasis using the selected features. The ROC curve results of the LightGBM model are shown in Fig. 3a. The ROC of the LightGBM model in the training, test, and test1 cohorts were 0.945 (95% CI 0.910–0.981), 0.892 (95% CI 0.813–0.971), and 0.908 (95% CI 0.836–0.980), respectively. The ROC of the combined model in the training, test, and test1 cohorts were 0.955 (95% CI 0.934–0.976), 0.835 (95% CI 0.739–0.931), and 0.918 (95% CI 0.856–0.981), respectively. Other performance parameters are presented in Table 2.

**Table 2:** Performance of models for predicting discrimination between breast cancer with bone metastasis and breast cancer without bone metastasis in training, test, and test1 cohorts

Model	Cohort	AUC (95% CI)	Accuracy	Sensitivity	Specificity	PPV	NPV	Precision	Recall	F1	Threshold
LightGBM	Training	0.945 (0.910–0.981)	0.870	0.850	0.889	0.879	0.862	0.879	0.850	0.864	0.455
	Test	0.889 (0.800–0.978)	0.811	0.923	0.704	0.750	0.905	0.750	0.923	0.828	0.379
	Test1	0.908 (0.836–0.980)	0.841	0.774	0.906	0.889	0.806	0.889	0.774	0.828	0.559

*AUC* Area under the curve; *PPV* Positive predictive value; *NPV* Negative predictive value; *F1* F1 Score

**Fig. 4:**
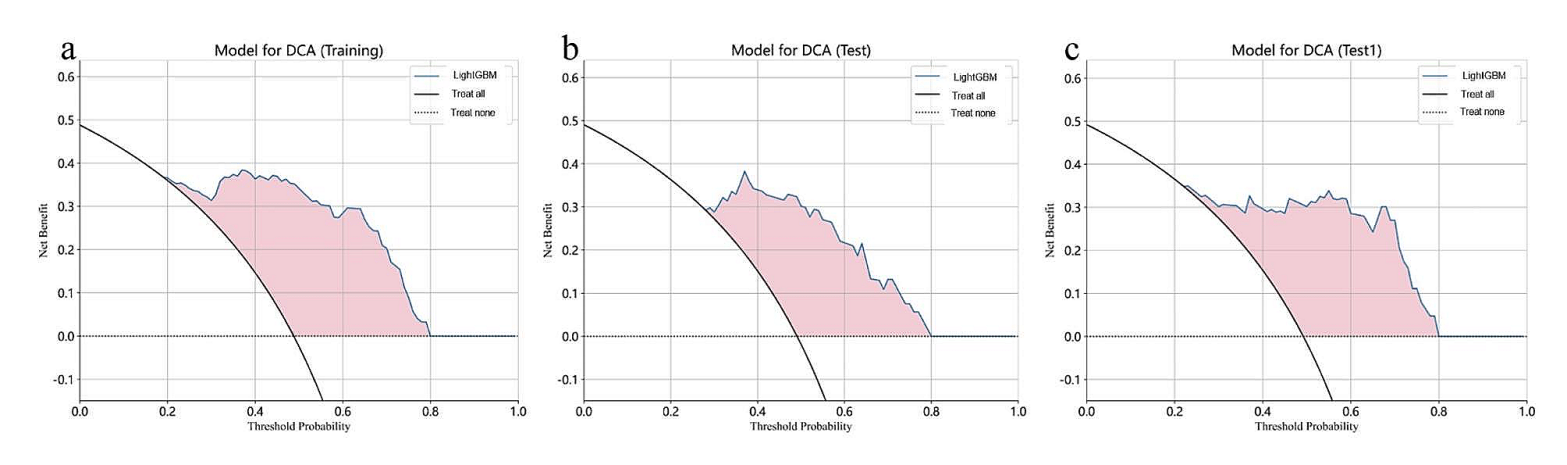
Clinical decision curves analysis (DCA) for the LightGBM models constructed in the training (**a**), test (**b**), and test1 (**c**) cohorts were demonstrated

The DCA curves of the LightGBM model in the training, test, and test1 cohorts are shown in Fig. 4. The results indicate that the LightGBM model demonstrates good net benefits in identifying breast cancer bone metastasis across all three cohorts.

### Feature importance analysis and logistic regression analysis

**Fig. 5:**
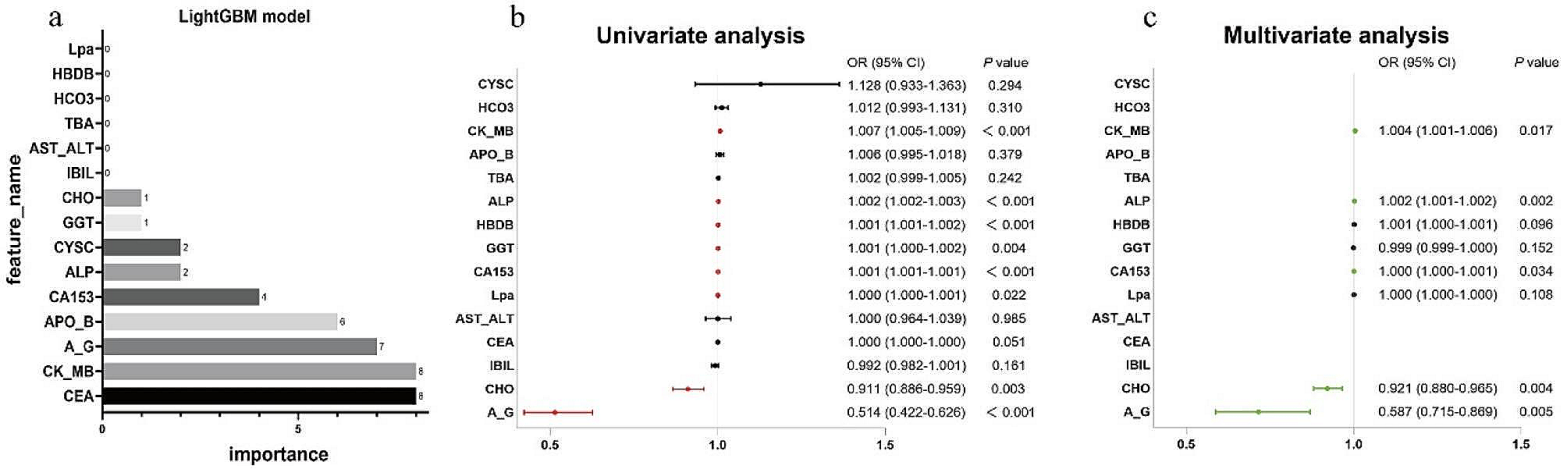
Feature importance analysis of LightGBM model (**a**) and univariate (**b**) and multivariate (**c**) logistic regression analysis of variables (features) involved in LightGBM model

To identify features crucial for predicting bone metastasis in the LightGBM model, feature importance analysis was conducted, as shown in Fig. 5a. The top 5 features with relatively high impact on the labels in the LightGBM model were CEA, creatine kinase, albumin/globulin ratio, apolipoprotein B, and CA153. Univariate and multivariate regression analyses were performed on the features involved in the model, with odds ratios and p-values displayed in Fig. 5b and c. In the univariate regression analysis, p-values of albumin-globulin ratio, total cholesterol, lipoprotein a, CA153, gamma-glutamyl transferase, α-HBDH, alkaline phosphatase, and creatine kinase were < 0.05, suggesting potential associations with breast cancer metastasis. Among these, lipoprotein a, CA153, gamma-glutamyl transferase, α-HBDH, alkaline phosphatase, and creatine kinase were positively correlated, while white blood cell count and total cholesterol were negatively correlated.

In the multivariate analysis, albumin-globulin ratio and total cholesterol had p-values < 0.05 and were negatively correlated. CK-MB, CA153, and alkaline phosphatase were positively correlated.

## Discussion

In this study, we utilized the LightGBM algorithm to construct a predictive model for identifying breast cancer patients with bone metastasis based on relatively easily accessible clinical blood biomarker features. The model demonstrated favorable performance in both internal and external testing cohorts. Our predictive model effectively distinguished breast cancer patients with bone metastasis from those without, providing clinicians with additional evidence to facilitate more efficient triage management in breast cancer diagnosis and treatment.

Most previous studies on predicting breast cancer distant metastasis have focused on assessing the risk of metastasis occurrence. Delpech et al. developed and validated nomograms for predicting bone metastasis in early-stage breast cancer patients based on clinical and pathological variables, with C-indexes of 0.69 and 0.73 in the training and validation cohorts, respectively [19]. Similarly, Xu et al. constructed nomograms for predicting bone metastasis in breast cancer patients based on clinical and pathological variables, with C-indexes of 0.714 and 0.705 in the training and validation cohorts, respectively [20]. Zhang et al. incorporated MRI and ultrasound features into prognostic nomograms for predicting distant metastasis in breast cancer, achieving C-indexes of 0.882 and 0.812 in the training and validation cohorts, respectively [21]. Additionally, Wang et al. utilized gene expression data from the National Center for Biotechnology Information Gene Expression Omnibus to construct prognostic nomograms for predicting lung metastasis risk in breast cancer, achieving C-indexes of 0.862 and 0.772 in the training and validation cohorts, respectively [22].

However, fewer predictive models have been developed specifically for diagnosing breast cancer distant metastasis. Wen-Cai et al. developed a web-based predictor using the XGBoost model to forecast the risk of bone metastasis in breast invasive ductal carcinoma patients based on factors such as diagnostic age, race, gender, grade, T/N staging, breast subtype, and marital status. The XGBoost model exhibited the best predictive performance among six different machine learning algorithms, with an AUC of 0.888, accuracy of 0.803, sensitivity of 0.801, and specificity of 0.837 [23]. Similarly, based on the Surveillance, Epidemiology, and End Results database, Xuguang et al. constructed diagnostic and prognostic models for breast cancer bone metastasis using the XGBoost algorithm, which achieved the highest accuracy (diagnostic model AUC = 0.98; prognostic model AUC = 0.88) [24]. However, these models often lack commonly available clinical indicators such as blood routine and biochemical parameters, which may limit their real-world applicability and require further validation.

This study represents the first attempt to construct a diagnostic predictive model for breast cancer bone metastasis using relatively easily accessible clinical blood biomarkers reflecting heart, liver, and kidney function. These biomarkers are typically part of routine admission tests for patients, providing real-time physiological information and offering cost-effective and easy-to-operate advantages compared to pathological examinations, imaging studies, or genetic tests. Additionally, our model underwent external validation at another research center and demonstrated satisfactory performance, with an AUC of 0.908. This external validation not only enhanced the credibility of our research findings but also demonstrated the model’s robustness and generalizability across different datasets.

In contrast to the relatively high-performing XGBoost model [25], this study employed the LightGBM machine learning algorithm. LightGBM exhibited greater flexibility and efficiency in feature processing and model construction, capable of handling complex nonlinear relationships better, thereby enhancing the model’s predictive accuracy and generalization capability. Despite achieving an AUC of over 0.9 in predicting breast cancer bone metastasis in our study, direct comparison of these AUC values with those of other models is not appropriate due to differences in variables and machine learning algorithms used. This diagnostic predictive model based on clinical blood biomarkers offers a novel and cost-effective approach for early detection of breast cancer bone metastasis. It not only contributes to improving personalized treatment management for breast cancer patients but also enhances the accuracy and efficiency of early intervention in clinical practice. Future research could further expand sample sizes and conduct multicenter validations to further verify the model’s robustness and broad applicability, thereby advancing its clinical implementation.

CK-MB was identified as one of the most important features in the LightGBM model prediction. As a creatine kinase isoenzyme, CK-MB exists mainly in the myocardium and skeletal muscle and has been found to be elevated in the serum of late-stage cancer patients compared to early-stage patients [26]. Previous studies have shown that serum CK-MB activity is significantly higher in patients with metastatic tumors compared to primary tumors [27]. However, further research is needed to elucidate why CK-MB elevation occurs in breast cancer patients with distant metastasis [26] and whether the elevated CK-MB originates from tumors or other sources [28]. α-HBDH, another important feature in our model, is an LDH isoenzyme that has been associated with prognosis in various malignant tumors [29–31]. In early breast cancer diagnosis, α-HBDH, CEA, and CA125 have been shown to have certain value when used in combination [32]. CA153, a common tumor marker, has predictive capabilities for breast cancer distant metastasis and was also identified as an important feature in our model [33].

Although we successfully constructed a predictive model for breast cancer bone metastasis using clinical blood biomarkers and demonstrated good predictive performance and external validation results, we still face several potential limitations and challenges. Firstly, we only focused on the most common type of breast cancer distant metastasis—bone metastasis. Thus, we did not consider other types of distant metastasis such as brain metastasis and post-treatment breast cancer metastasis [34]. Secondly, although our external validation set originates from different medical centers within the same geographical region, these data still have limitations. Similar patient demographics and treatment protocols may restrict the model’s generalizability globally. Future research should incorporate more extensive multi-center, geographically diverse external validation sets to further validate the model’s performance across diverse populations and enhance its generalizability and reliability. Additionally, while we selected relatively accessible clinical blood biomarkers as input variables for the predictive model, the specificity and sensitivity of these biomarkers may not fully cover all complex scenarios of breast cancer bone metastasis. In clinical practice, it may be necessary to combine more biomarkers or other clinical features to further optimize the model’s predictive ability. Furthermore, although the LightGBM algorithm performs well in handling complex nonlinear relationships, its sensitivity to data quality and feature selection needs attention. The quality of data, standardization, and feature selection significantly impact model performance. Future research needs to further optimize these aspects to enhance the stability and reliability of the model. Lastly, with advances in technology and medical research, new biomarkers and technologies continue to emerge, which may pose new challenges and opportunities for the construction and application of existing models. Therefore, continuous technological innovation and data updates are crucial for the ongoing optimization and widespread application of the model.

## Conclusions

In conclusion, this study successfully developed and validated artificial intelligence clinical models and comprehensive models for predicting breast cancer bone metastasis based on clinical blood biomarkers. Particularly, the LightGBM model exhibited high accuracy and potential clinical utility in predicting and identifying breast cancer bone metastasis. In China’s healthcare system, patients with advanced cancer stages are often referred to economically developed regions for treatment, while underdeveloped regions may experience delayed diagnosis due to a lack of early cancer screening. Therefore, the model has the potential to mitigate disease misdiagnosis caused by a lack of imaging technology in underdeveloped regions and improve the clinical decision-making skills of primary care physicians, thereby providing patients with more timely treatment. Similarly, in developed regions, the model can reduce the demand for expensive or invasive imaging techniques. This study highlights the prospect of using easily accessible clinical blood biomarkers for developing artificial intelligence predictive tools.

### Electronic supplementary material

Below is the link to the electronic supplementary material.

Supplementary Material 1

## Data Availability

All datasets generated for this study are included in the article/Supplementary Material.
