# Supplementary material for: Development and validation of an artificial intelligence model for predicting de novo distant bone metastasis in breast cancer: a dual-center study
Source: BMC Womens Health. 2024 Aug 5;24:442. doi: 10.1186/s12905-024-03264-z (PMC11299401; doi:10.1186/s12905-024-03264-z)
Supplement: Supplementary file 1 — Supplementary Material 1 [file 12905_2024_3264_MOESM1_ESM.docx]

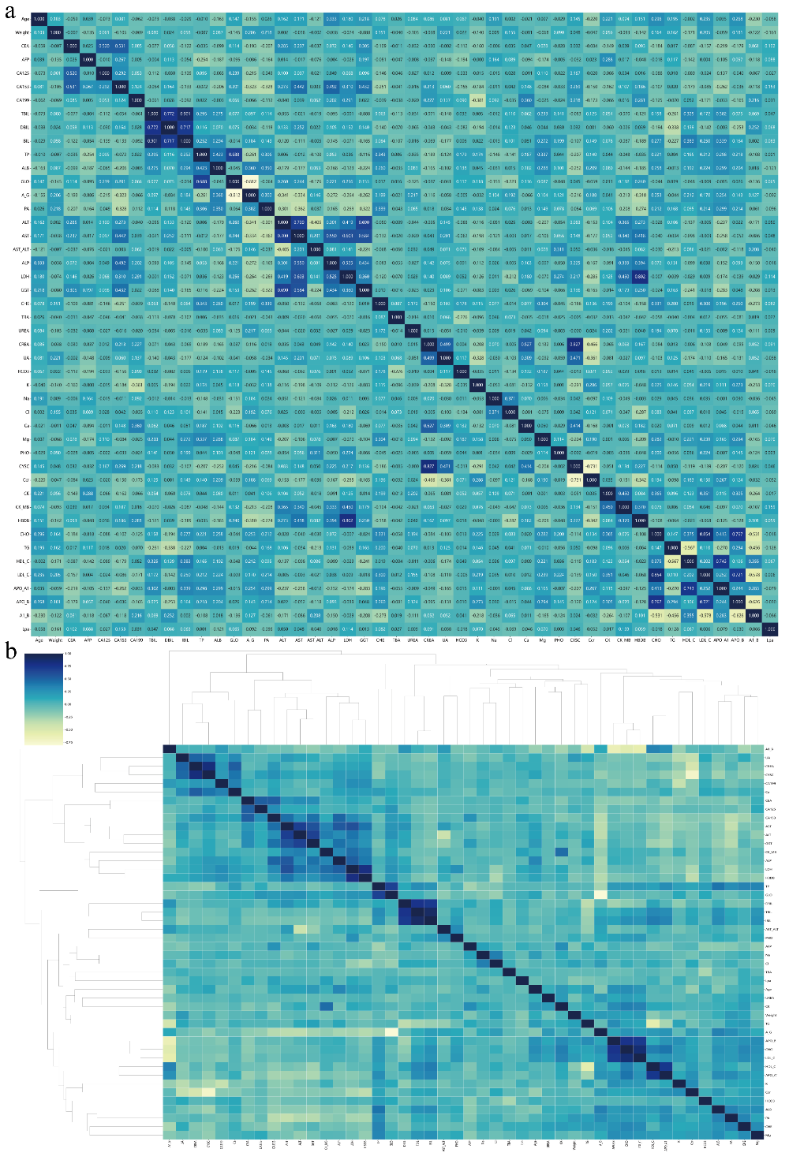


Supplementary Figure 1a-b The correlation analysis of features in the form of a heatmap, illustrating pairwise correlations between features. The areas where features intersect show correlation coefficients ranging from 0 to 1.
